# Spatial and temporal evolution of ecological vulnerability based on vulnerability scoring diagram model in Shennongjia, China

**DOI:** 10.1038/s41598-022-09205-w

**Published:** 2022-03-25

**Authors:** Jia-shuo Cao, Yu-qi Yang, Zheng-yu Deng, Yuan-dong Hu

**Affiliations:** 1 College of Landscape Architecture, Northeast Forestry University, Harbin, 150040 China; 2 Key Laboratory for Garden Plant Germplasm Development & Landscape Eco-Restoration in Cold Regions of Heilongjiang Province, Harbin, 150040 China; 3 School of Design, The University of Western Australia, Perth, 6009 Australia

**Keywords:** Ecological modelling, Environmental impact

## Abstract

Shennongjia is one of the most important ecological function areas and ecologically vulnerable zones in the world. With the rapid development of social economies, especially tourism, the ecological environment of Shennongjia has experienced profound changes. Exploring the characteristics and changing trends of ecological environment in Shennongjia will help to analyze the causes of the damage to the ecological environment, and build a vulnerability analysis framework with multi-scale, multi-element, multi-flow, and multi-circulation characteristics, which provides an effective research paradigm and analysis tool for the study of regional ecological vulnerability. With the support of RS and GIS technology, this study uses spatial principal component analysis (SPCA) and the vulnerability scoring diagram (VSD) model to comprehensively and quantitatively analyze the spatial and temporal evolution characteristics and driving forces of ecological vulnerability in Shennongjia from 1996 to 2018. The VSD model was selected to decompose the vulnerability into three components of "exposure-sensitivity-adaptation", and 16 indicators were selected to construct an ecological vulnerability evaluation system in Shennongjia, and the evaluation data were organized in a progressive and detailed way. (1) During the study period, the overall ecological vulnerability of Shennongjia is in a mild vulnerability level, exhibiting differentiation characteristics of high in the northeast and low in the southwest. High vulnerability zones are mainly distributed in the main towns and roads. (2) The risk of ecological vulnerability of the entire region presents the characteristics of continuous decline. (3) Land-use types, population density, and vegetation coverage are the main factors driving the evolution of ecological vulnerability. (4) A high level of coupling coordination exists between ecological vulnerability and landscape patterns. Analyses of the ecological vulnerability of Shennongjia shows that the entire region is in a mild vulnerability level. The extreme vulnerability risk of the ecological environment shows polarization. The evolution of ecological environment in Shennongjia is the result of the interaction between human activities and natural environment. This study offers an effective way to assess ecological vulnerability and provides some strategies and guidance for improving ecological security.

## Introduction

Since the appearance of human beings, the relationship between man and the earth has existed as a new operating mechanism^1^. With the deepening of economic and social development, the carrying capacity and buffer capacity of the natural environment has been tested^2^. The disordered development strategy in the early stage of this relationship caused serious environmental pollution and waste of resources, and regional ecosystems are now on the verge of degradation^3,4^. Indeed, in recent years, the destruction of the natural environment has become increasingly apparent, including climate warming^5^, land desertification^6^, biodiversity reduction^7^, and other major ecological vulnerability problems, and the trend of globalization and intergenerational change has gradually emerged^8^.

Ecological vulnerability concerns the structure and function of an ecosystem. It is a comprehensive assessment of the degree of disturbance damage, the degree of system damage, and the ability of system restoration^9^. Recently, research on ecological vulnerability has attracted great interest from scholars at home and abroad, and plentiful research results have been achieved in theory and empirical aspects^10^. The research objects involve many typical ecological functional areas, such as soil and water conservation functional areas, agro-pastoral ecotone, lakes, wetlands, rivers, forests, arid areas, mountainous areas, and resource-based cities. The research contents are continually deepening, including the response of ecological vulnerability and sensitivity to climate change in macro-regions^11^, the spatial differentiation of the pattern and process of ecological vulnerable areas^12,13^, and the formation mechanism of ecological vulnerability^14^. Vulnerability assessment has become a novel research paradigm in current global ecological environment changes and sustainable development^15^, as well as an effective tool to analyze the processes and mechanisms of man-land interaction.

Shennongjia is located in the transition zone between the second and third step of terrain in China. It is one of the most important ecological function areas and ecological vulnerable zones in the world, and has gained substantial attention due to its superior natural ecological endowment. Meanwhile, it has the only well-preserved subtropical forest ecosystem in the middle latitudes and is one of the world’s biodiverse area. Shennongjia is a gene pool of global significance, known as the "Green Miracle". The area includes various ecosystems such as forests, shrubs, meadows and wetlands, which have important climate regulation, and soil and water conservation values. However, from the 1960s to the 1980s, due to excessive logging, a large number of natural landscapes were replaced by man-made places, which inevitably caused damage to the regional ecological environment and further increased ecological vulnerability^16^. Facing the grim reality, local governments began to explore green and sustainable economic development models. Since the 1990s, ecological protection projects in the Shennongjia area started to be implemented one after another.

In recent decades, the typical ecological regions including Shennongjia have been strongly disturbed by human activities, showing great fluctuation in both space and time. However, there is no relevant research on the ecological vulnerability evaluation of Shennongjia. Therefore, this study hopes explore the symbiotic relationship between economic development and nature protection in ecologically vulnerable areas, in order to provide typical cases and theoretical support for the field of ecological vulnerability assessment.

The aim of this study is to reveal the evolution of the ecological environment in Shennongjia, and to build a sustainable development model for ecologically vulnerable areas. Hopefully in the future, can improve the regional landscape and environmental quality, and can maintain regional ecological security and stability.

## Materials and methods

### Description of the study area

**Figure 1:**
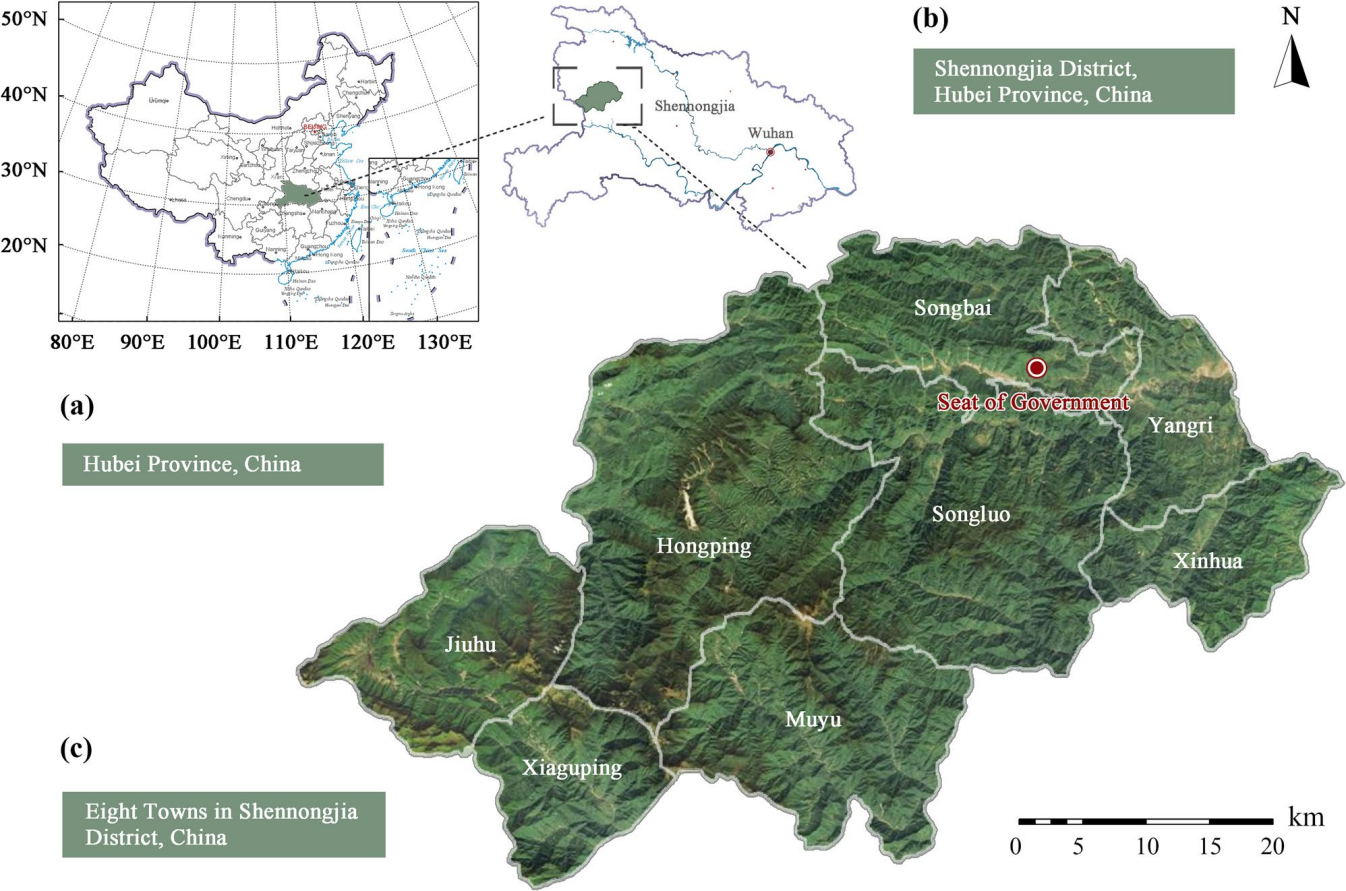
Location map of Shennongjia.

Shennongjia is located in the mountainous area of northwest Hubei (Fig. 1), between 109°56′–110°58′ E, 31°15′–31°57′ N, with a total area of 3215.80 km^2^. The southwest is dominated by mountains in the east–west direction. The highest peak of Shennongjia is 3105.40 m, which is also the highest point in central China^17^. Shennongjia is the intersection zone of east–west flora and the transition zone of north–south flora in China. It possesses the only well-preserved subtropical forest ecosystem in the middle latitudes of the world, as well as the world’s most abundant biodiversity^18,19^.

### Data sources and preprocessing treatments

This study involves 17 data indicators (Table 1). Among them, the four indicators of surface relief, slope, aspect, and water system distribution are all based on data of 2018 due to their minimal change in a short time-span. The other indicators use relevant data of 1996, 2007, and 2018.

**Table 1:** Description and source of evaluation indexes.

Indicator	Method and explanation	Data sources
Land-use types	125/38, 126/38 of Landsat 5 TM/Landsat 8 OLI image interpretation in 1996, 2007, 2018 Five categories: forest land; grassland; cultivated land; construction land; and water bodies	Website of the United States Geological Survey (USGS) (http://glovis.usgs.gov/)
Slope	Extracted from the digital elevation model (DEM)	Website of USGS (http://glovis.usgs.gov/)
Aspect	Extracted from the digital elevation model (DEM)	
Surface relief	Maximum elevation value − minimum elevation value in unit area	
Vegetation coverage	Mixed pixel decomposing model	Website of USGS (http://glovis.usgs.gov/)
Average annual temperature	Spatial interpolation combined with regression equation calculation and interpolation residuals	Website of the China Meteorological Administration (http://data.cma.cn/)
Annual precipitation		
Population density	Population/land area	Shennongjia Bureau of Statistics
Local fiscal revenue per capita	Local fiscal revenue/population	
Water distribution	Mapping of field research and historical data	Shennongjia Bureau of Water Resources and Lakes
Surface water resources	Monitoring statistics	
Quality of surface water	Monitoring statistics	
Industrial wastewater discharge	Monitoring statistics	
Domestic sewage discharge	Monitoring statistics	
Annual tourist reception	According to Shennongjia tourism management report statistics	Shennongjia Bureau of Culture and Tourism
National park policy	Organized according to field research and data collection	Shennongjia National Park Administration
Nature reserve policy		

The land-use data of Shennongjia is interpreted based on Landsat remote sensing images. The spatial resolution of the images is 30 m. According to the national standard of land use status classification (GB/T 21010-2017) and the purpose of this study, the land in Shennongjia is divided into five categories: forest land; grassland; construction land; cultivated land; and water bodies.

Due to the differences of data-source types and spatial accuracy of different evaluation indexes, inverse distance weighted interpolation (IDW) was used for spatial deterministic interpolation of certain statistical data with the support of the ArcGIS software platform (the raster size of spatial data was defined as 30 m × 30 m) to realize spatial localization.

### Index system of ecological vulnerability assessment

**Figure 2:**
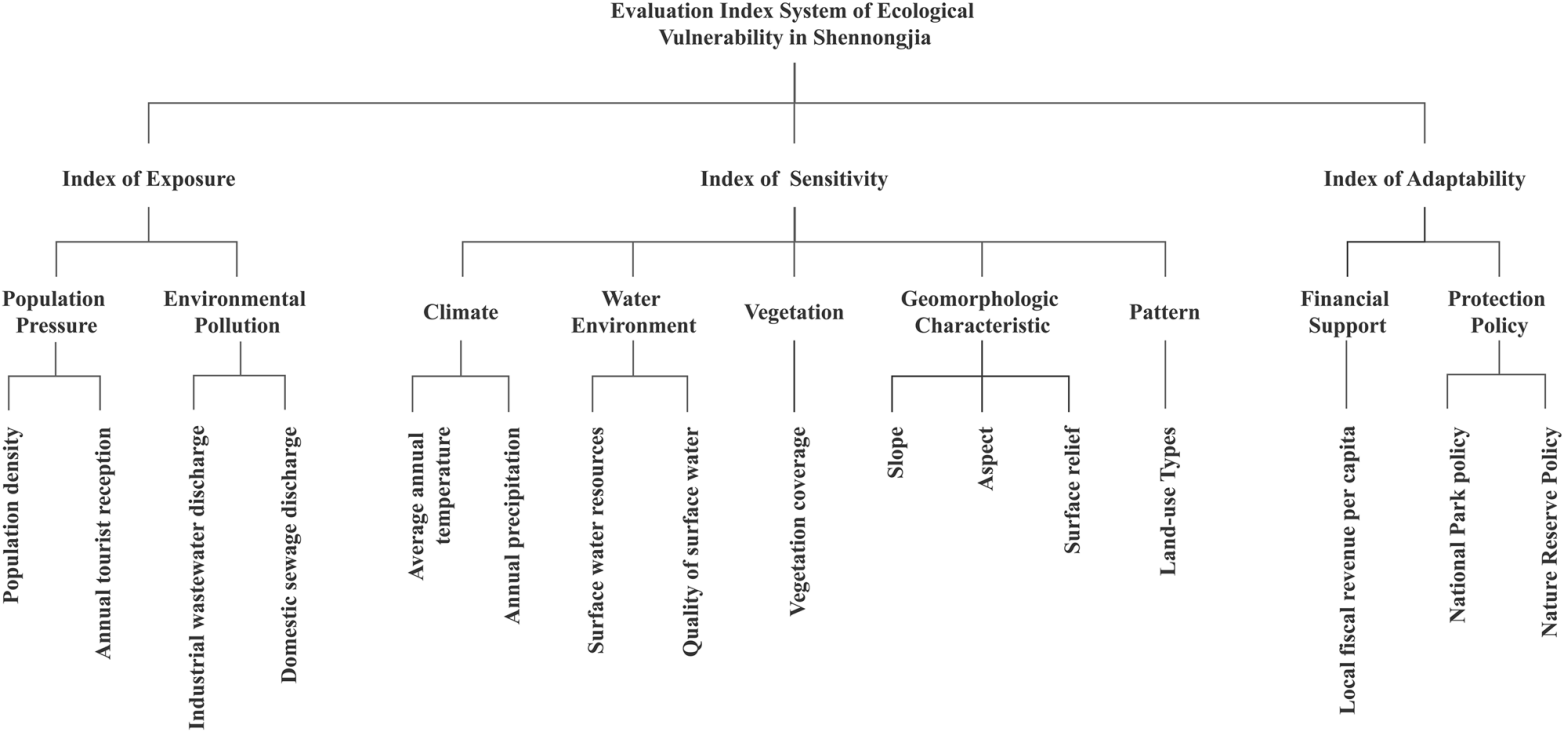
Ecological vulnerability evaluation index system of Shennongjia.

In this study, the VSD model was used. Combined with previous research results^20^, through field investigation and historical data processing, the following 16 indicators were selected from three main aspects of exposure, sensitivity and adaptability, and the ecological vulnerability evaluation system of Shennongjia was constructed (Fig. 2).

Indexes of exposure can reflect the degree of which the ecosystem is affected by external disturbance or stress^21^. Population density and annual tourist reception are selected to reflect the threat of population pressure on the ecosystem. Industrial wastewater and residential wastewater discharge are chosen to indicate ecological vulnerability results from environmental pollution.

Indexes of sensitivity can reflect the response of part or the whole ecosystem to changes of the natural environment and human activities, and indicate the probability of ecological imbalance and other environmental problems in a particular region^22^. Ecosystems with high sensitivity have a high risk of ecological problems and are the focus of restoration and protection; systems with low sensitivity are not susceptible to changes due to disturbance and are suitable for rational development. According to the actual situation of Shennongjia, climate characteristics, water environment quality, topography, vegetation status, and land use were chosen as important evaluation factors of ecological sensitivity.

Indexes of adaptability reflect the system's ability to adjust and cope with changes or disturbances of internal and external conditions, and indicate the measures and countermeasures that human beings take to deal with many ecological problems^23^. Local fiscal revenue per capita can reflect regional economic development and, to a certain extent, it can represent the financial investment capacity of human society for ecological construction and ecological protection projects. The delimitation of nature reserves and national parks directly indicates the implementation of regional protection policies.

### Index standardization

Due to the differences of dimensions, orders of magnitude and positive and negative directions of the above evaluation indexes, and in order to eliminate such possible impacts on data analysis, this study uses the range method and the hierarchical assignment method to standardize the original data of each index^24^. The calculation formula is as follows^25^:

1 $$\text{Positive} \; \text{evaluation} \; \text{index}{:} \;\;Y_{i} = \frac{{X_{i} - X_{{{\text{min}}}} }}{{X_{{{\text{max}}}} - {\text{X}}_{{{\text{min}}}} }} \times 10$$

2 $$\text{Negative} \; \text{evaluation} \; \text{index}{:} \;\;Y_{i} = \frac{{X_{{{\text{max}}}} - X_{{\text{i}}} }}{{X_{{{\text{max}}}} - X_{{{\text{min}}}} }} \times 10$$

where *Y*_*i*_ is the value of the ith indicator after standardized calculation, its range is 0–10, and the larger is *Y*_*i*_, the higher is the ecological vulnerability of the region and the more vulnerable is the ecosystem to external disturbance and damage; *X*_*i*_ is the original value of the *i*th index; *X*_*max*_ is the maximum of the original value of the *i*th index; and *Xmin* is the minimum of the original value of the *i*th index.

As a qualitative index, land-use types need to be classified and quantified ^46,48^. According to previous studies^47^), and combined with the actual situation of Shennongjia, the standardized assignment of each land-use type was performed (Table 2).

**Table 2:** Standardized grading assignment of land-use types.

Land-use types	Forest land	Grassland	Cultivated land	Construction land	Water bodies
Assignment	2	4	6	8	2

### Spatial principal component analysis

Principal component analysis (PCA) is a commonly-used multivariate statistical analysis method. This data dimension reduction algorithm is generally applied to feature extraction^26^. Spatial principal component analysis (SPCA) is based on the support of the ArcGIS software platform, which extends the method of PCA to two-dimensional space^27,28^, so that many related complex spatial information data can be transformed into a few unrelated comprehensive indicators. The visual PCA of evaluation objects can then be completed^29,30^.

Using the PCA module tool of ArcGIS, the spatial SPCA of 16 evaluation indexes in four levels is performed. Taking the cumulative contribution rate of principal components reaching more than 90% as the standard, the first six principal components (Table 3) are selected to replace the original 16 variables for analysis, in order to achieve data dimensionality reduction.

**Table 3:** Eigenvalue, contribution rate, and accumulated contribution rate of each principal component.

Year	Principal component coefficient	Principal component					
		PC1	PC2	PC3	PC4	PC5	PC6
1996	Eigenvalues λ	1.681	0.567	0.429	0.227	0.207	0.112
	Contribution rate (%)	47.17	15.90	12.04	6.36	5.80	3.13
	Accumulated contribution rate (%)	47.17	63.07	75.11	81.47	87.27	90.40
2007	Eigenvalues λ	1.864	0.861	0.435	0.258	0.186	0.149
	Contribution rate (%)	46.13	21.32	10.77	6.39	4.61	3.69
	Accumulated contribution rate (%)	46.13	67.45	78.22	84.61	89.22	92.92
2018	Eigenvalues λ	2.049	0.439	0.402	0.303	0.239	0.135
	Contribution rate (%)	52.52	11.25	10.29	7.77	6.13	3.45
	Accumulated contribution rate (%)	52.52	63.77	74.06	81.84	87.97	91.42

Ecological environmental vulnerability is an essential indicator to measure the level of regional ecological vulnerability. According to the extracted principal components, the formula for the ecological vulnerability index is as follows^31^:

3 $$EVI = r_{1} Y_{1} + r_{2} Y_{2} + r_{3} Y_{3} + \cdots + r_{n} Y_{n}$$

where *EVI* is the ecological vulnerability index; Yi is the *i*th principal component; and *r*_*i*_ is the corresponding contribution rate of the *i*th principal component. The calculation formula of the contribution rate is:

4 $$r_{i} = \frac{{\lambda_{i} }}{{\sum\nolimits_{i = 1}^{n} {\lambda_{i} } }}$$

where *r*_*i*_ is the corresponding contribution rate of the *i*th principal component; and *λ*_*i*_ is the eigenvalue of the *i*th principal component.

Overall, the larger is the ecological vulnerability index, the more vulnerable is the ecological environment and the worse is the system stability. In contrast, the smaller is the ecological vulnerability index, the higher is the stability of the ecosystem.

In order to solve the problem of comparability of ecological vulnerability assessment results in different years, and to analyze and measure them more clearly and intuitively, the EVI index is standardized, the calculation formula of which is as follows^32^:

5 $$SVI_{{\text{i}}} = \frac{{EVI_{i} - EVI_{\min } }}{{EVI_{\max } - EVI_{\min } }} \times 10$$

where *SVI*_*i*_ is the standardized value of the ecological vulnerability index in the *i*th year, and its variation range is 0–10; *EVI*_*i*_ is the actual value of the ecological vulnerability index in the *i*th year; *EVI*_*max*_ is the maximum of the ecological vulnerability index over many years; and *EVI*_*min*_ is the minimum value of the ecological vulnerability index.

### Ecological vulnerability classification

According to the characteristics of the ecological environment in Shennongjia and related research results at home and abroad, the ecological vulnerability classification standard of Shennongjia was established. According to the differences of ecological vulnerability in the unit area, the study area was divided into five grades by using the equal difference classification method, which are: micro degree, mild degree, moderate degree, severe degree, and extreme vulnerability areas (Table 4). Based on the analysis of different levels of vulnerability and characteristics, this paper discusses the measures of ecological environment protection and the suitability scope of future national park development planning.

**Table 4:** Ecological vulnerability grading standard of Shennongjia.

Degree	Level	Standardized value	Characteristics of ecological vulnerability
Micro vulnerability	I	< 2.0	The structure and function of the ecosystem are reasonable and complete, the ecosystem is stable, the ability of resisting external interference and self-recovery is strong, and there is no ecological abnormality
Mild vulnerability	II	2.0–4.0	The structure and function of the ecosystem are relatively complete, the ecosystem is relatively stable, the ability to resist external interference and self-recovery is strong, and there are potential ecological anomalies
Moderate vulnerability	III	4.0–6.0	The structure and function of the ecosystem can be maintained, the ecosystem is relatively unstable, it is sensitive to external interference, it possesses weak self-recovery ability, and there are a few ecological anomalies
Severe vulnerability	IV	6.0–8.0	The structure and function of the ecosystem are defective, the ecosystem is unstable, it is sensitive to external interference, it is difficult to recover after damage, and there are many ecological anomalies
Extreme vulnerability	V	≥ 8.0	The structure and function of the ecosystem are seriously degraded, the ecosystem is extremely unstable, it is extremely sensitive to external interference, and it is very difficult to recover after being damaged

### Regression fitting analysis

Taking the spatial data of ecological vulnerability index as the unit, the least square method is used to carry out linear regression analysis, and the slope of the fitting line is obtained to characterize the change trend of ecological vulnerability in the study period. The slope calculation formula shows as follows^33^:

6 $$K = \frac{{n \times \sum\nolimits_{i = 1}^{n} {i \times } EVI_{i} - \left( {\sum\nolimits_{i = 1}^{n} i } \right)\left( {\sum\nolimits_{i = 1}^{n} {EVI_{i} } } \right)}}{{n \times \sum\nolimits_{i = 1}^{n} {i^{2} } - \left( {\sum\nolimits_{i = 1}^{n} i } \right)^{2} }}$$

where *K* is the slope; *n* is the number of years; and *EVI*_*i*_ is the ecological vulnerability index of the *i*th year. If the slope value is positive, the ecological vulnerability index increases, and the regional ecological vulnerability increases; if the slope value is negative, the ecological vulnerability index decreases, and the regional ecological vulnerability decreases.

### Comprehensive ecological vulnerability index

In order to better express the quantitative characteristics of ecological vulnerability and determine the overall state of Shennongjia in the form of intuitive quantification, the comprehensive ecological vulnerability index (CEVI) was constructed to calculate the ecological vulnerability. The formula shows as follows:

7 $$CEVI = \sum\limits_{i = 1}^{n} {L_{i} } \times \frac{{A_{i} }}{S}$$

where *L*_*i*_ is the grade value of level *i* vulnerability; *A*_*i*_ is the area of level *i* vulnerability; and *S* is the total area of the study area.

### Correlation and contribution analysis

As the number of patches (NP), largest patch index (LPI), aggregation index (AI), landscape division index (DIVISION) and Shannon diversity index (SHDI) are more sensitive to the change of landscape structure, and can fully reflect the fragmentation and diversity of regional landscape pattern, these five indexes are selected as the representative of the landscape pattern index^34,35^. Coupling analysis with the ecological vulnerability index was conducted to identify the contribution of landscape pattern to vulnerability.

In multiple linear regression, if the dependent variable *Y* is linearly correlated with the independent variable *X*_*i*_* (i* = *1, 2, ……, k, k is the number of independent variables)*, the formula for calculating the sum of squares *P*_*i*_ of the partial regression of the independent variable *X*_*i*_ to *Y* is:

8 $$U = ({\hat{\text{y}}} - {\overline{\text{y}}})^{2}$$

9 $$P_{{\text{i}}} = U - U_{{\text{i}}} (i = 1,2, \ldots \ldots ,{\text{k}})$$

where u is the sum of the regression square of the whole model; *U*_*i*_ is the sum of the regression square of *k-1* linear regression equation after Xi is excluded; and *P*_*i*_ is the sum of the partial regression square of *X*_*i*_ of each independent variable. Overall, the larger is the *P*_*i*_ value, the greater is the contribution of *X*_*i*_ to the regression square sum *U*. The formula for calculating the contribution rate is as follows:

10 $$S = {{P_{{\text{i}}} } / {\sum\limits_{i = 1}^{k} {P_{{\text{i}}} } }} \times 100\%$$

where *S* is the contribution rate, and is the ratio of *P*_*i*_ to the cumulative value of all *P*_*i*_.

## Results

### Spatial and temporal distribution of ecological vulnerability

**Figure 3:**
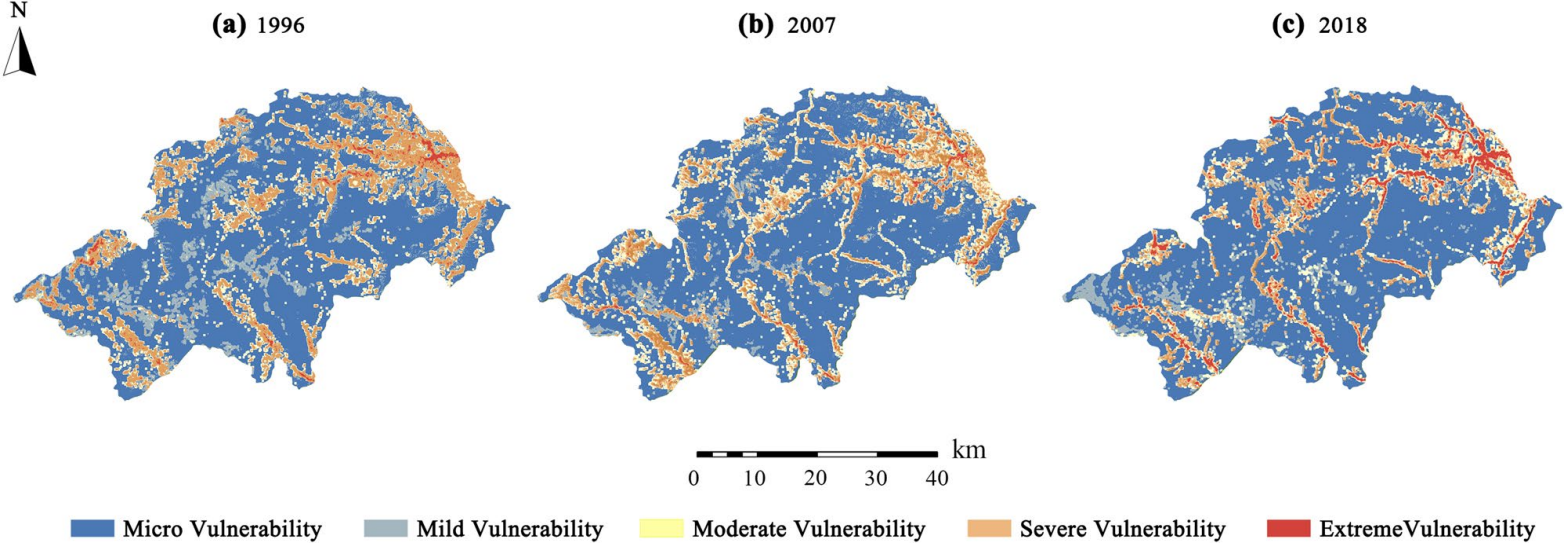
Spatial and temporal distribution of ecological vulnerability in Shennongjia. Spatial and temporal distribution of ecological vulnerability for (**a**) 1996, (**b**) 2007, (**c**) 2018 in Shennongjia, China.

Based on the SPCA model, the temporal and spatial distribution of ecological vulnerability in Shennongjia is obtained, as shown in Fig. 3. From 1996 to 2018, the area of micro vulnerability areas continued to increase and occupied a dominant position. Moreover, their distribution pattern tended to be gradually integrated, indicating that the structure and function of the ecosystem in most areas of Shennongjia were relatively complete, and in a healthy and stable state. However, the ecological environment of the severely vulnerable areas in the northeast, south and southwest of Shennongjia is in a trend of continuous deterioration, and the risk of extreme vulnerability is gradually emerging. From the spatial distribution of ecological vulnerability in 2018, it can be seen that the extremely vulnerable areas have increased significantly, and exhibit a dense and continuous distribution trend in some areas, accompanied by the development of rapid urbanization and highway traffic construction. There are also high-risk ecological vulnerable zones and the extremely vulnerability areas.

**Figure 4:**
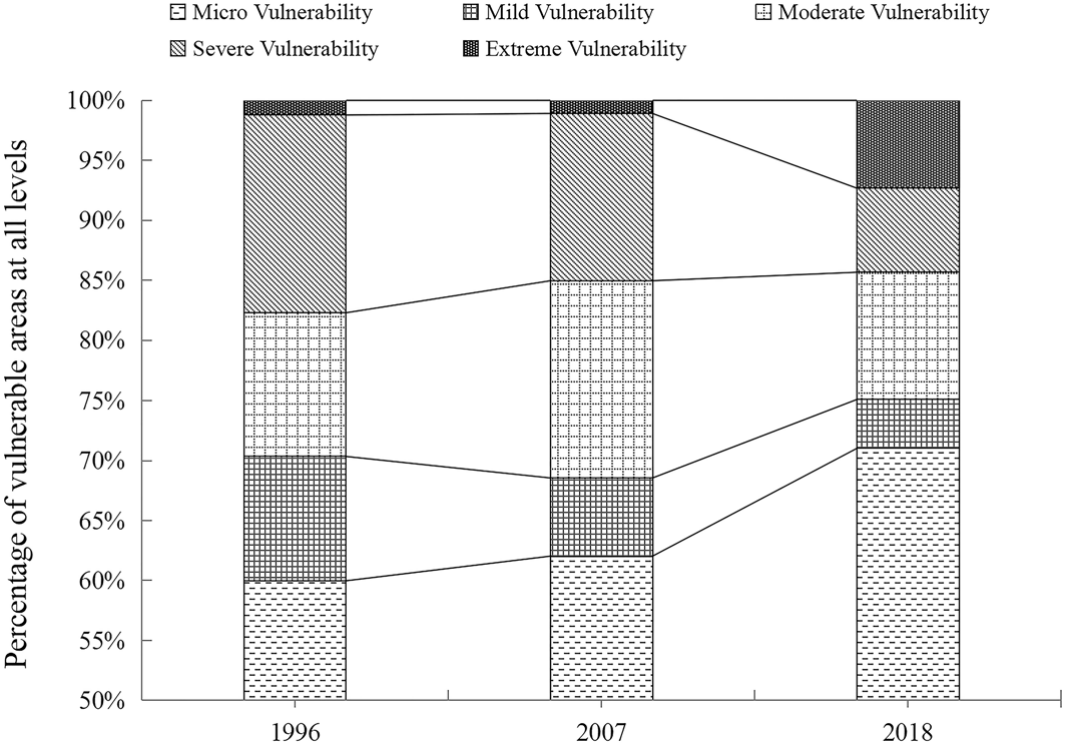
Proportion of the area of vulnerable districts at all levels in Shennongjia.

It can be seen from the area proportion of different levels of vulnerable areas (Fig. 4) that the area proportion of micro and extremely vulnerable areas increased significantly. Specifically, the area proportion of micro vulnerable areas increased from 59.98% in 1996 to 71.02% in 2018, while the area proportion of extremely vulnerable areas increased from 1.23% in 1996 to 7.32% in 2018. This shows that the ecological vulnerability of Shennongjia exhibits a significant two-level differentiation trend.

### Dynamic change of ecological vulnerability

**Figure 5:**
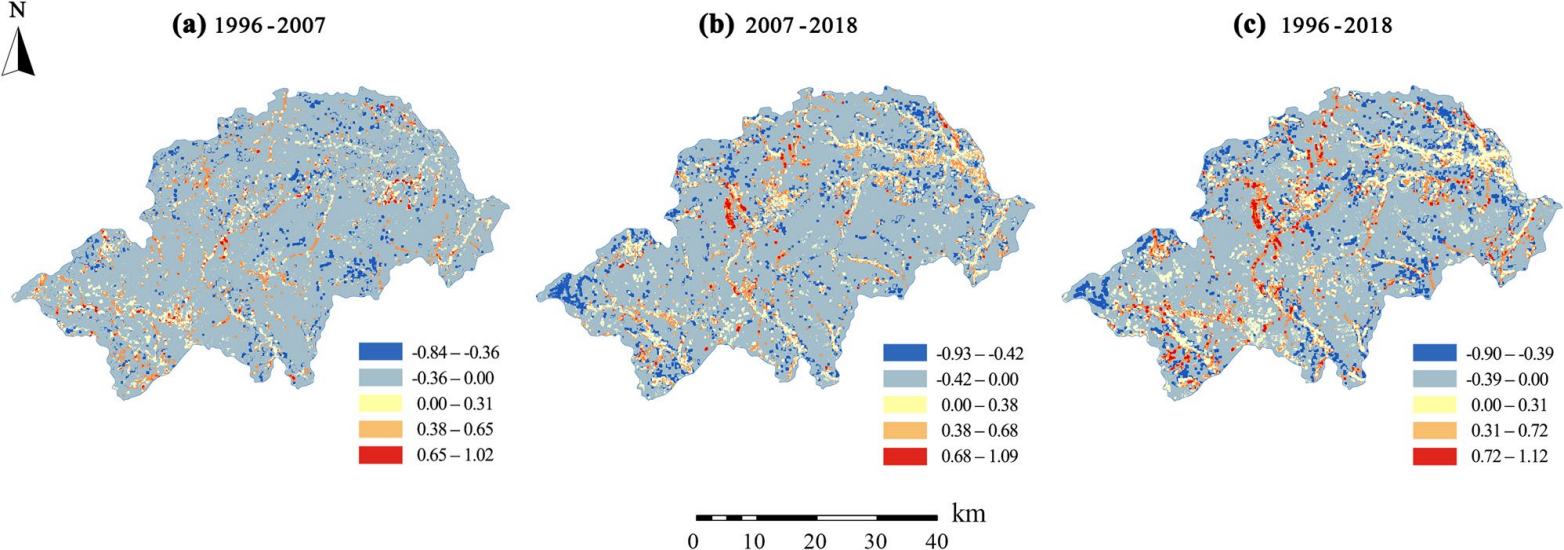
Dynamic changes of ecological vulnerability in Shennongjia. Changes in the ecological vulnerability of Shennongjia in different periods: (**a**) 1996–2007, (**b**) 2007–2018, (**c**) 1996–2018.

During the study period, the areas with a positive fitting slope account for more than 90% of the total area of the study area, which indicates that the overall vulnerability of Shennongjia presents a downward trend. According to the natural discontinuity point method, the dynamic change results of ecological vulnerability in Shennongjia are divided into five levels (Fig. 5), in order to discern the spatial angle more intuitively and clearly. It can be seen that the ecological vulnerability of most regions exhibits a decreasing trend, while the ecological vulnerability of certain regions increases.

From 1996 to 2007, whether the spatial distribution trend of ecological vulnerability increased or decreased is not obvious. However, from 2007 to 2018, the areas with significantly increased ecological vulnerability were concentrated in Yangri and Songbai in the northeast and near the Hongping airport in Shennongjia in the midwest. During this same time period, in the areas around the main urban areas and along the roads that were seriously disturbed by human activities, ecological vulnerability also exhibited a decreasing trend.

### Change trend of comprehensive ecological vulnerability index

#### Annual change of the comprehensive ecological vulnerability index

**Figure 6:**
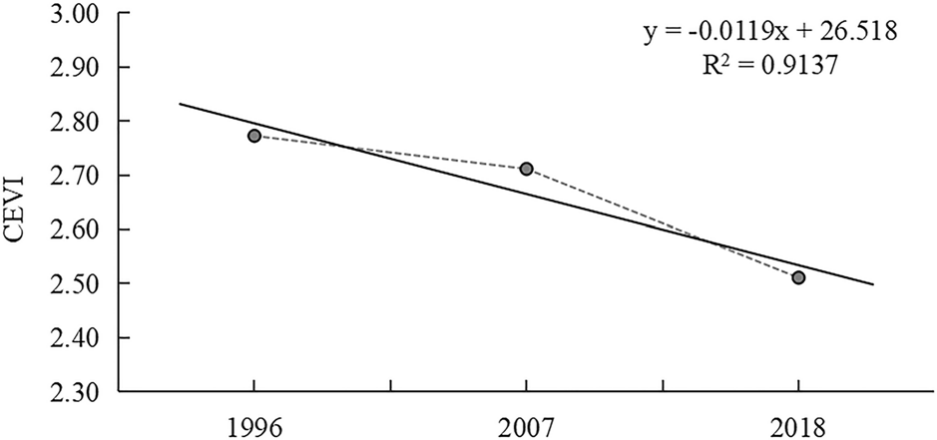
Annual change of the comprehensive ecological vulnerability index. CEVI, comprehensive ecological vulnerability index.

The results of the comprehensive ecological vulnerability index of 1996, 2007, and 2018 are 2.77, 2.71, and 2.51, respectively. From the annual change of the ecological vulnerability index in Shennongjia (Fig. 6), it can be seen that the ecological vulnerability of Shennongjia showed a downward trend from 1996 to 2018, and the stability and health of the ecosystem were improved overall.

Among them, the decline of ecological vulnerability is relatively small from 1996 to 2007, which may be ascribed to the preliminary implementation of restrictive policies, such as banning logging and returning farmland to forest, which reduced ecological exposure factors, such as illegal logging and deforestation. From 2007 to 2018, the comprehensive index of ecological vulnerability in Shennongjia decreased significantly, which is mainly due to the designation of national nature reserves and the implementation of various ecological protection projects^36^. While reducing the exposed ecological disturbance, it simultaneously markedly improved the adaptability of the ecosystem, and further reduced the overall ecological vulnerability of the region.

#### Changes of the comprehensive ecological vulnerability Index in different towns

**Figure 7:**
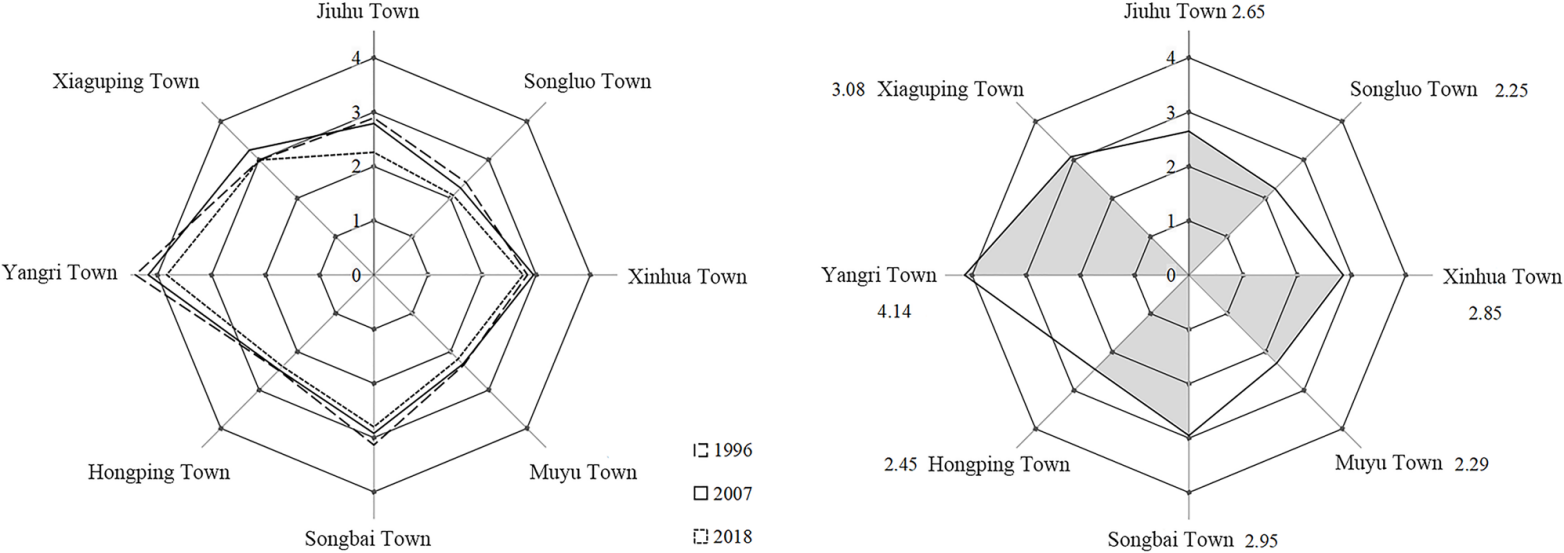
Radar chart of the comprehensive ecological vulnerability index of towns.

According to the comprehensive index of ecological vulnerability of eight towns in the Shennongjia (Table 5, Fig. 7), the ecological vulnerability difference of each town is obvious. In 2018, the comprehensive index of ecological vulnerability of each town is lower than that in 1996 and 2007. The results show that the average value of CEVI is, from high to low, Yangri, Xiaguping, Songbai, Xinhua, Jiuhu, Hongping, Muyu, and Songluo. The maximum value of the CEVI appeared in Yangri in 1996, and the minimum value occurred in Songluo in 2018.

**Table 5:** Comprehensive ecological vulnerability index of towns.

Year	Songbai	Muyu	Yangri	Hongping	Xinhua	Songluo	Jiuhu	Xiaguping	Total
1996	3.13	2.35	4.41	2.49	2.84	2.41	2.89	2.99	2.77
2007	2.92	2.32	4.17	2.48	2.95	2.27	2.79	3.25	2.71
2018	2.80	2.20	3.83	2.39	2.76	2.08	2.26	2.99	2.51
Mean	2.95	2.29	4.14	2.45	2.85	2.25	2.65	3.08	2.66

### Driving factors of spatial and temporal evolution of ecological vulnerability

The formation and evolution of ecological vulnerability in Shennongjia constitutes a dynamic process, which is the result of interactions of human and natural factors. Based on the principle of SPCA of ecological vulnerability, the transformed principal components are extracted, and the rotated factor load matrix is obtained to reflect the different effects of various factors on the evaluation results. Each principal component possesses a different ability to explain the original index factors, but it has similar rules in the first four principal components (Table 6). The cumulative contribution rate of the first four principal components in the three groups of data reached more than 80%, which can reflect the information of most factors, and thus it has good representativeness.

**Table 6:** Principal component loading and score.

Index	1996				2007				2018			
	PC1	PC2	PC3	PC4	PC1	PC2	PC3	PC4	PC1	PC2	PC3	PC4
C1	− 0.06	0.88^a^	− 0.05	0.11	− 0.06	0.70^a^	− 0.05	0.02	− 0.02	0.94^a^	− 0.31	− 0.16
C2	− 0.04	0.11	− 0.03	− 0.04	− 0.03	0.00	0.09	− 0.04	− 0.03	0.04	0.10	0.06
C3	0.26	− 0.15	0.02	0.06	0.25	0.01	− 0.15	− 0.01	0.20	− 0.05	− 0.13	− 0.07
C4	0.02	0.08	− 0.01	− 0.21	0.02	0.00	0.11	− 0.26	0.34	0.00	− 0.05	0.05
C5	0.07	0.01	0.02	− 0.08	0.13	0.02	− 0.11	− 0.14	0.31	0.13	− 0.05	0.02
C6	0.15	0.23	0.04	− 0.28	0.15	− 0.01	− 0.31	− 0.27	0.14	− 0.09	− 0.25	− 0.10
C7	0.14	0.05	0.01	0.05	0.15	0.01	− 0.05	0.01	0.14	− 0.01	− 0.05	0.01
C8	− 0.04	0.13	− 0.03	− 0.01	− 0.06	0.52	− 0.05	0.02	− 0.03	0.05	0.13	0.08
C9	0.72^a^	0.43	0.98^a^	0.19	0.70^a^	0.08	0.86^a^	0.34	0.69^a^	0.05	0.81^a^	− 0.53
C10	0.26	− 0.02	0.01	0.16	0.30	0.03	− 0.03	0.11	− 0.08	− 0.17	− 0.44	− 0.06
C11	0.25	− 0.17	0.03	0.06	0.25	0.01	− 0.19	− 0.04	0.26	− 0.05	− 0.18	− 0.05
C12	0.41	− 0.31	0.05	0.12	0.38	0.01	− 0.32	− 0.03	0.31	− 0.10	− 0.27	− 0.17
C13	− 0.11	− 0.32	− 0.03	0.55^a^	− 0.10	− 0.05	− 0.38	0.64^a^	0.01	0.03	0.13	0.78^a^
C14	0.05	− 0.05	0.01	− 0.01	0.07	0.00	− 0.08	− 0.04	0.06	− 0.02	− 0.06	− 0.01
C15	0.11	0.35	0.02	− 0.50	0.10	0.06	0.33	− 0.45	0.08	0.17	0.41	0.07
C16	− 0.20	0.48	− 0.17	0.44	− 0.23	0.01	0.39	0.31	− 0.15	0.13	0.38	0.12

^a^Represents that the contribution of this ingredient is high.

Among the first principal component and the third principal component, the contribution of land-use type index (C9) is higher; in the second principal component, the contribution of population density (C1) is higher; among the fourth principal components, the contribution of vegetation coverage (C13) is higher. Moreover, the contribution of other factors in different years and main components is dissimilar.

#### The influence of land-use type on ecological vulnerability

Whether due to natural or human factors, the original properties of the ecosystem are altered by changing the surface cover. Therefore, land-use type is an important factor affecting regional ecological vulnerability. The difference of surface cover leads to the difference of ecological community, and then produces varied ecological environmental benefits. Forest land is the most important land-use type in the study area, and the ecological vulnerability of the distribution area is mainly micro degree and light. However, consider the important ecological value of the forest ecosystem, attention should be given to its vulnerability. The ecological vulnerability of the construction land is mainly severe and extreme, which is largely due to the expansion of construction land, which destroys the original ecological structure and ecological community. Furthermore, a large number of manmade patches replace natural patches in the construction land, and biodiversity decreases, leading to the decline of the stability of ecological structures and the increase of vulnerability.

#### The influence of population density on ecological vulnerability

Population density is one of the most direct exposure factors in the vulnerability of ecological environments. Population density is generally higher than that in high area, and it is also a region with a developed economy and high urbanization. In these areas, human activities are frequent, which usually impart a negative disturbance to the natural environment, including the rapid expansion of cultivated land and construction land area, as well as high discharge of production and domestic wastewater waste, which has caused great pressure on the ecological environment, leading to a significant increase in ecological vulnerability.

#### The influence of vegetation cover on ecological vulnerability

From 1996 to 2018, the vegetation coverage of the Shennongjia exhibited an overall upward trend, which is of positive significance to the reduction of the vulnerability of the ecosystem. Vegetation, as the main body of the land ecosystem, maintains the balance of ecological environment through interactions with climate, landform, and soil^37^. Extant literature shows that the change of vegetation coverage is an major factor of regional ecological environment change, and has a clear indication function for the change of regional ecological environment^38^. The spatial distribution trend of ecological vulnerability in the Shennongjia is markedly similar to that of vegetation coverage. The ecological vulnerability of regions with higher vegetation coverage is lower, exhibiting a significant negative correlation. In the Shennongjia, the change of vegetation coverage is also obviously influenced by human factors.

### Contribution of landscape pattern index to ecological vulnerability

**Figure 8:**
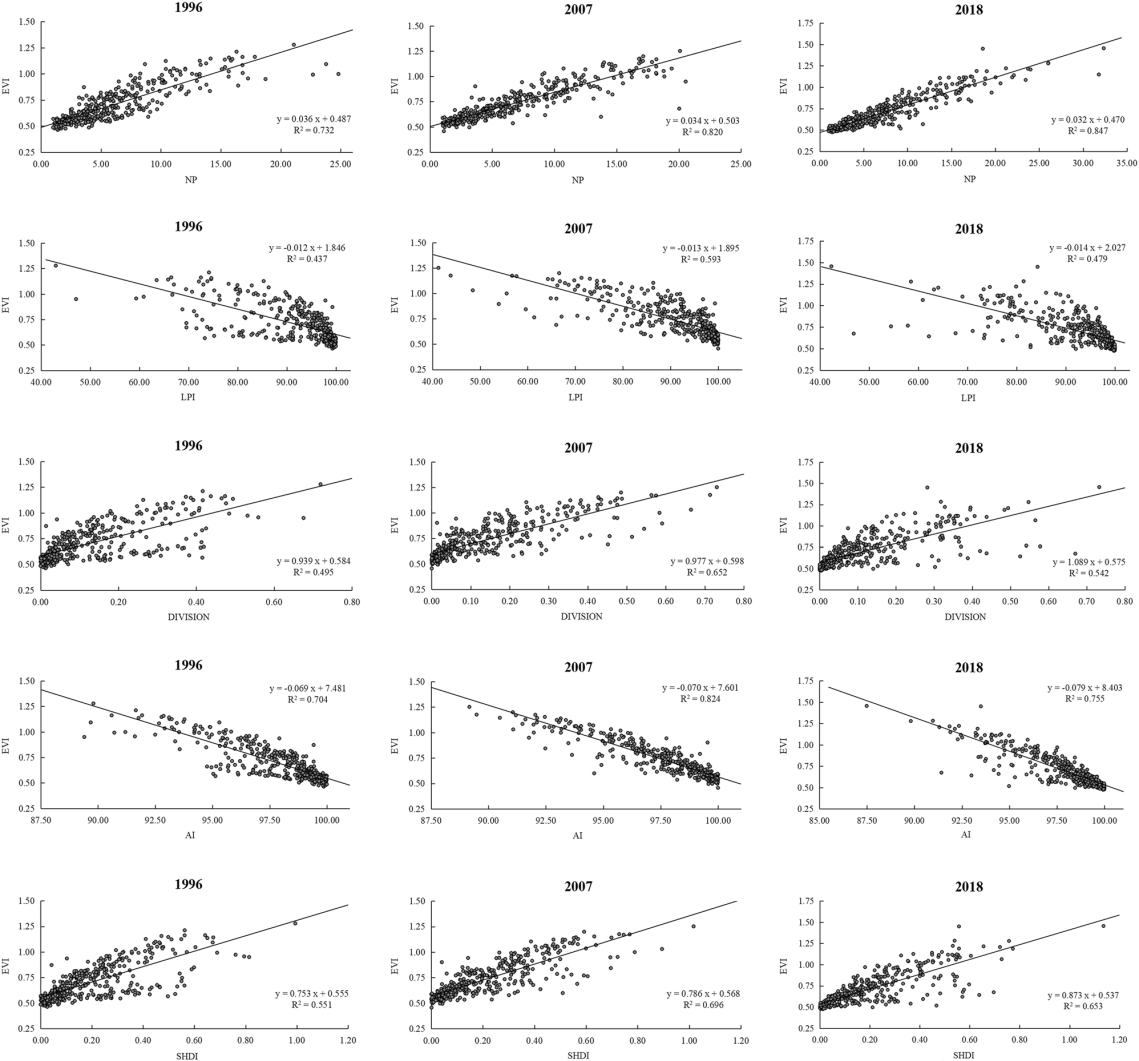
Scatter plot of linear regression of landscape pattern index and ecological vulnerability index. EVI, ecological vulnerability index.

The spatial distribution of each index in Shennongjia have been obtained from previous studies^47^. From the unary linear regression analysis, in the years of 1996, 2007 and 2018, the NP, LPI, AI, DIVISION and SHDI are all significantly correlated with the ecological vulnerability index (Fig. 8).

In the case of different independent variable combinations in 1996, 2007 and 2018, the multiple regression relationship between the independent variable and the dependent variable of each group is significantly correlated, and the multiple linear regression equation of the full model is obtained as follows:

$$1996{:}\;\;{\text{ Y}} = 6.443 + 0.014{\text{X}}_{1} + 0.006{\text{X}}_{2} - 0.038{\text{X}}_{3} - 0.066{\text{X}}_{4} + 0.058{\text{X}}_{5}$$

$$2007{:}\;\;{\text{ Y}} = 4.497 + 0.016{\text{X}}_{1} + 0.007{\text{X}}_{2} + 0.793{\text{X}}_{3} - 0.047{\text{X}}_{4} - 0.305{\text{X}}_{5}$$

$$2018{:}\;\;{\text{ Y}} = - 1.980 + 0.037{\text{X}}_{1} + 0.006{\text{X}}_{2} + 0.703{\text{X}}_{3} + 0.019{\text{X}}_{4} - 0.123{\text{X}}_{5}$$

The contribution rate of landscape pattern index to ecological vulnerability in different years of 1996, 2007, and 2018 is shown in Table 7. The contribution of AI and NP to ecological vulnerability in 1996 was high; the contribution of NP and AI to ecological vulnerability was higher in 2007; and the NP in 2018 had the highest contribution to ecological vulnerability, reaching 95.77%.

**Table 7:** Contribution of the landscape pattern index to the ecological vulnerability index.

Year	Combination of independent variables	Partial regression sum of squares	Contribution rate (%)
1996	X_1_, X_2_, X_3_, X_4_, X_5_	0.20988	–
	X_1_, X_2_, X_3_, X_4_	0.00038 (X_5_)	0.18%
	X_1_, X_2_, X_3_, X_5_	0.13735 (X_4_)	65.44%
	X_1_, X_2_, X_4_, X_5_	0.00002 (X_3_)	0.01%
	X_1_, X_3_, X_4_, X_5_	0.00856 (X_2_)	4.08%
	X_2_, X_3_, X_4_, X_5_	0.06357 (X_1_)	30.29%
2007	X_1_, X_2_, X_3_, X_4_, X_5_	10.09184	–
	X_1_, X_2_, X_3_, X_4_	0.03261 (X_5_)	5.35%
	X_1_, X_2_, X_3_, X_5_	0.12079 (X_4_)	38.05%
	X_1_, X_2_, X_4_, X_5_	0.01645 (X_3_)	10.38%
	X_1_, X_3_, X_4_, X_5_	0.01380 (X_2_)	4.40%
	X_2_, X_3_, X_4_, X_5_	0.13250 (X_1_)	41.82%
2018	X_1_, X_2_, X_3_, X_4_, X_5_	12.35475	–
	X_1_, X_2_, X_3_, X_4_	0.00363 (X_5_)	0.46%
	X_1_, X_2_, X_3_, X_5_	0.01168 (X_4_)	1.37%
	X_1_, X_2_, X_4_, X_5_	0.01226 (X_3_)	1.49%
	X_1_, X_3_, X_4_, X_5_	0.00820 (X_2_)	0.91%
	X_2_, X_3_, X_4_, X_5_	0.83736 (X_1_)	95.77%

X_1_—NP, X_2_—LPI, X_3_—DIVISION, X_4_—AI, X_5_—SHDI.

Based on the analysis results from 1996 to 2018, the contribution of NP and AI to ecological vulnerability is relatively high. The main reason for this is that the forest coverage rate of Shennongjia is as high as 91%. Specifically, with the forest as the landscape matrix, the NP is small and the connectivity between patches is high, showing a trend of aggregation. The degree of landscape fragmentation is relatively low and decreases annually, and ecological vulnerability decreases with the decrease of the degree of landscape fragmentation, Therefore, the impact of NP and AI on ecological vulnerability is highly significant.

The AI and ecological vulnerability index always exhibit a significant negative correlation in the study period. In the 1996 research results, the contribution of AI to ecological vulnerability is the most obvious. Combined with the spatial distribution of ecological vulnerability, it can be seen that most of the severe and extremely vulnerable areas are distributed in areas with low AI. Most of them are the distribution areas of artificial patches, such as rural living areas, airports, tourism centers, etc., which are obviously disturbed by human activities, resulting in low connectivity among various landscape types, which greatly reduces the aggregation degree of landscape and increases regional vulnerability.

There is also a significant positive correlation between the NP and the ecological vulnerability index. This is especially the case in 2018, when the contribution of the NP to ecological vulnerability is as high as 95.77%, which is mainly attributable to the urbanization construction of Songbai town in Shennongjia. Combined with the land-use structure map, it can be seen that the number of construction land patches in the northeast region increased sharply. In this process, the renewal of patches aggravates the degree of landscape fragmentation and plays a key role in the aggravation of regional vulnerability risk.

Although the impact of LPI, SHDI and DIVISION on ecological vulnerability always exists, the contribution is not very significant. Among them, SHDI contributed 10.38% in 2007, which was more sensitive to the unbalanced distribution of each patch type. In areas with high SHDI, landscape heterogeneity is high, the ecological pattern is unstable, and ecological vulnerability increases.

## Discussion

### Spatial and temporal differentiation characteristics and driving forces of ecological vulnerability

From 1996 to 2018, the ecological vulnerability of Shennongjia was generally slightly vulnerable, and it decreased gradually. Although the ecological environment was stable and good, there were high-risk ecological problems in some extremely vulnerable areas, which must be considered.

From the spatial dimension, the ecological vulnerability of Shennongjia shows the distribution characteristics of high in the northeast and low in the southwest. The extremely vulnerable and severely vulnerable areas are primarily distributed in the main towns and roads in Shennongjia. These areas are characterized by rapid urbanization and development, dense populations, and more social and economic activities, especially housing land. The expansion of construction land for public facilities also caused significant damage and pollution to the ecological environment, thus forming a highly vulnerable area, which is consistent with the conclusion described in previous relevant research on "low habitat quality and high type of human activities"^39^. The distribution of micro vulnerability areas and mild vulnerability areas is broad, with the proportion of the area exceeding 70% of the whole Shennongjia. Most of these areas are elevated and far away from the main rivers, but the situation of disturbance and damage caused by human settlements is avoided, and the vegetation is well covered. Indeed, the ecological environment is good. It is worth noting that in recent years, there has been increasing demand for Shennongjia eco-tourism. The remote mountainous areas are highly valued by tourists because of their original natural features. As tourism resources, they have been gradually developed. However, with the increase of human activities, their vulnerability to life has increased risk.

Concerning the time dimension, the risk of ecological vulnerability in the study area presents the characteristics of continuous decline. The main reasons for this are as follows:

Firstly, the comprehensive implementation of ecological protection projects makes the ecological environment of most regions effectively protected and restored. In terms of forest protection, natural forest protection, conversion of farmland to forest, three green projects, and the establishment of a National Forest Park are the main measures. The wetland protection measures primarily include the establishment of the Dajiu Lake National Wetland Park, and the protection of endangered wild animals^40,41^.

Secondly, the implementation of ecological restoration projects in highly vulnerable areas has eased the contradiction of local human land systems. From the change trend of ecological vulnerability in 2007–2018, the ecological vulnerability of the main town areas and areas along the roads decreased significantly. In recent years, many ecological restoration projects have been implemented in these areas which were disturbed by high-level people, such as ecological space planning in cities and towns, the cultivation of characteristic economic forests and landscape forests, and the construction of landscape belts along the highway, gradually reducing the risk of regional vulnerability^42^.

Thirdly, urbanization in Shennongjia in recent years has caused much of the population to move out from natural reserved areas to urban areas^43^. Whether it is due to the impact of the ecological protection immigration policy or spontaneous behavior, the migration of a large population objectively reduces the disturbance to the original habitat and improves the efficiency of ecological protection. Fourth, the concept of ecological protection is deeply rooted in people's priorities, concerning the promotion of ecological civilization construction. The Shennongjia government focuses on cultivating public ecological ethics, and popularizing a series of laws and regulations, including forest law and wildlife protection legislation, in order to improve awareness of environmental protection laws and establish a social norm of ecological protection^44^.

### Ecological environment protection strategy in Shennongjia

According to the analysis of the land use and ecological vulnerability, the following problems are found in Shennongjia:

The spatial distribution of ecological vulnerability is significantly different, and the ecology in local high-vulnerability areas needs to be restored urgently. Due to the predatory development and unscientific use of natural resources in the early stage, the locations of the main urban areas of each township, along the roads and major scenic spots have become typical high-vulnerability areas, and the self-sustaining and recovery capabilities of local ecosystems have been significantly reduced.

Road construction cuts the original ecological corridor and hinders the operation of ecological flow. With the advancement of urbanization and the development of tourism in the Shennongjia, the road network divided the landscape types to a certain extent, resulting in fragmentation of the area, and ecological gaps appeared in the original continuous ecological corridor network. These ecological gaps increase the resistance of the landscape, greatly reduce the connectivity of the landscape, and pose a serious threat to the safety of biological migration, resulting in an increased risk of ecological vulnerability.

The contradiction between regional ecological protection and regional development has become increasingly prominent, and there is a lack of ecological space layer control. The contradiction between ecological protection and economic development has existed for a long time in Shennongjia. Due to the lack of scientific zoning and ecological space layer control, the protection of ecological source areas is under great pressure.

Guided by the above problems, we believe that the optimization of landscape ecological pattern and the upgrading of the ecological environment by classification and division will become the main strategies for ecological protection in Shennongjia.

#### Optimization of landscape ecological pattern

Aiming at the health of the ecosystem, landscape management in Shennongjia is optimized by using the theory and method of landscape ecology, in order to realize reasonable land-use layout and scientific management, and to ensure ecological safety of the region^45^. In the future, we should consider the separation of some artificial landscape types to ensure the flow of ecological energy. For instance, the functional integration of fine patches can realize the aggregation development of homogeneous plaques. The reuse of idle wasteland should also be strengthened to improve the efficiency of patch utilization. Moreover, the scale of artificial corridor construction should be controlled, which will reduce the interference of human activities on habitat connectivity. Ecological corridors should also be formed, and biodiversity protection networks should be established.

#### The upgrading of the ecological environment by classification and division

**Figure 9:**
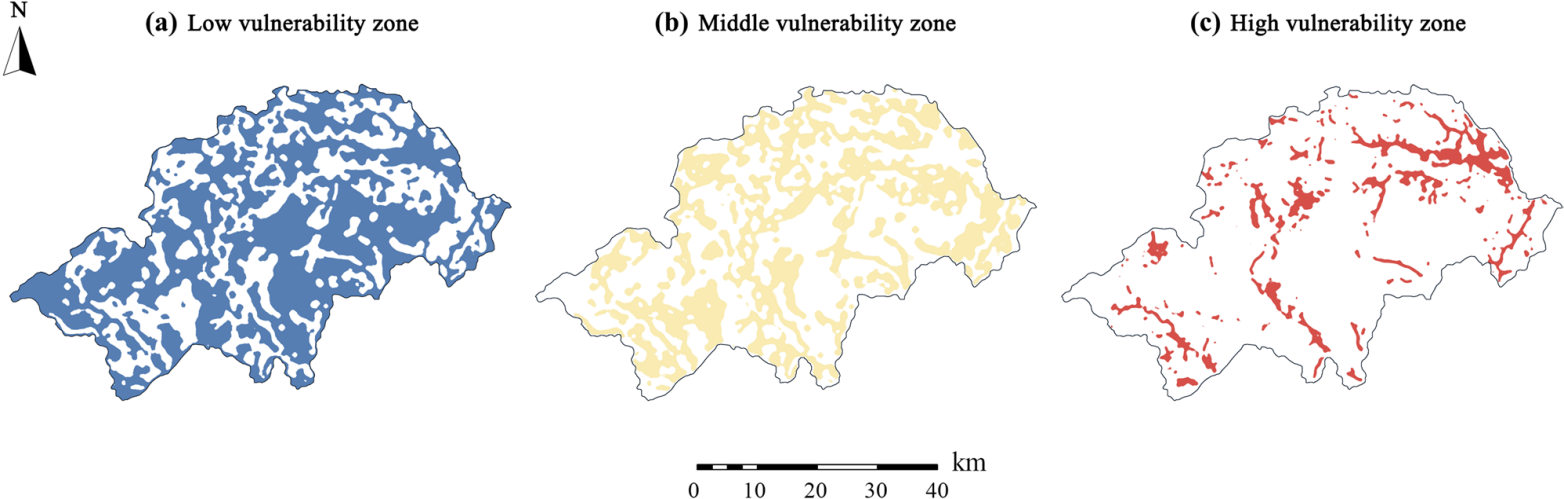
Schematic diagram of ecological environment improvement. (**a**) Low vulnerability zone mainly includes the micro and Mild vulnerability areas in the evaluation results. (**b**) Middle vulnerability zone is the transitional zone, which is mainly composed of the surrounding area of the ecological source or the low-impedance area. (**c**) High vulnerability zone mainly includes the severe and extreme vulnerability areas in the evaluation results.

Based on the differences of ecosystem composition and the causes of vulnerability, regional vulnerability is divided into three levels: low, medium, and high (Fig. 9). It is suggested that the strategy of classified partition protection and promotion be adopted. The targeted suggestions are as follows:

The main goal of ecological protection in low vulnerability zone is to maintain the long-term stability of the regional ecosystem. Most of the ecological resources in Shennongjia are distributed in low vulnerability zone, such as the north subtropical evergreen broad-leaved forest, Dajiu Lake wetland, and Sichuan snub-nosed monkey nature reserve. For low vulnerability zone, ecological protection red lines should be drawn to reduce the development intensity of ecologically-sensitive areas. Taking the ecological source area as the core, the scope of the surrounding buffer area should be scientifically established, existing projects should be strictly controlled within the scope, ecological constraints should be proposed, the scope of ecological protection should be expanded, and regional circle control should be implemented.

The main goal of ecological protection in middle vulnerability zone is control and improvement. On the one hand, it is necessary to build ecological security barriers to curb the spread of vulnerability factors. On the other hand, it is also necessary to repair the ecological environment of middle vulnerability zone to enhance the stability of the regional ecosystem. For the protection of middle vulnerability zone, focus must be given to boundary repair of production and living land, and vegetation coverage of the eroded area should be compensated through artificial intervention, in order to prevent ecological interference and erosion. Moreover, management and protection of ecological resources must be strengthened in middle vulnerability zone, investment in vegetation restoration in the central part of Shennongjia should be increased, and regional vegetation coverage should be improved.

The main goal of ecological protection in high vulnerability zone is restoration and reconstruction. Human subjective initiatives must be developed and implemented, ecological restoration and reconstruction projects should be carried out, continuous deterioration of the ecological environment should be markedly slowed, and the degree of regional vulnerability should be reduced. Specific protection strategies include promoting ecological restoration projects and improving the ecological compensation system, adjusting the industrial structure, and promoting harmonious development of the ecological environment and social economy. We should also rationally arrange the green space system and augment the construction of urban and rural green infrastructure.

## Conclusions

From 1996 to 2018, the ecological vulnerability of Shennongjia was in a light degree, the risk of extreme vulnerability in some areas appeared gradually, and the ecological environment was polarized. Regional ecological vulnerability shows the characteristics of high and low southwest in northeast China, among which the extremely vulnerable and severe vulnerable areas are primarily distributed in the main towns and towns along the highways in Shennongjia. The formation and development of ecological vulnerability in Shennongjia constitutes a dynamic process. The types of land use, population density, and vegetation coverage are the main driving factors of ecological vulnerability evolution. Ecological vulnerability and landscape pattern are highly correlated, among which the number of patches and aggregation index contribute greatly to ecological vulnerability.

This study is novel in three aspects. Firstly, based on a variety of quantitative remote sensing inversion theories and methods, the surface parameters can be extracted quickly and accurately, and the SPCA and moving window method can be used to realize the visualization of factor indicators. Through the classification of the results, we can grasp the distribution and evolution characteristics of ecological vulnerability from the spatial level. Secondly, the CEVI is constructed to express the overall ecological vulnerability state in an intuitive and quantitative form. Thirdly, this study provides pragmatic theoretical and technical guidance for reducing the risk of regional ecological environment vulnerability and improving ecological security. According to the results, in the future, ecological environment protection in Shennongjia should be carried out from the two aspects of landscape ecological pattern optimization and ecological classification division promotion. Indeed, coordinating the relationship between ecological environment protection, tourism resources development, and regional economic development will be the key to achieve sustainable development of Shennongjia.

There are also some drawbacks and future perspectives of the work. Some of the data used in this study are limited by data sources. It reflects in two aspects. On the one hand, the spatial resolution of Landsat 5 and Landsat 8 data are both 30 m, which are medium-resolution remote sensing images. Therefore, the land use classification, topography, vegetation cover and other data obtained by interpretation are limited in accuracy. On the other hand, some indicators of evaluation are based on townships, so the results are inevitably affected by the boundaries of administrative regions. Therefore, higher-resolution remote sensing images and more accurate spatial data can be considered for future research to obtain more accurate results. In the study of ecological vulnerability in Shennongjia, 16 evaluation indicators were selected for evaluation, but the factors causing ecological vulnerability are extremely complex. It is difficult to form a recognized and unified evaluation index system in the actual research process, and should be further improved in future research. This study aims to propose ecological protection and restoration strategies for Shennongjia. However, the restoration of ecological environment requires detailed and continuous research. Long-term monitoring should take place after the implementation of specific measures for further improvement.

## Data Availability

Data is available at Open Science Framework: Cao Jiashuo. 2022. "Spatial and Temporal Evolution of Ecological Vulnerability Based on Vulnerability Scoring Diagram Model in Shennongjia, China" OSF. https://osf.io/tpmg4/?view_only=18a78e7efdb14d07802c93ddfd3c260a.
